# GW0742 activates miR‐17‐5p and inhibits TXNIP/NLRP3‐mediated inflammation after hypoxic‐ischaemic injury in rats and in PC12 cells

**DOI:** 10.1111/jcmm.15698

**Published:** 2020-10-09

**Authors:** Marcin Gamdzyk, Desislava Met Doycheva, Ruiqing Kang, Hong Tang, Zackary D. Travis, Jiping Tang, John H. Zhang

**Affiliations:** ^1^ Department of Physiology and Pharmacology Basic Sciences School of Medicine Loma Linda University Loma Linda CA USA; ^2^ Department of Anesthesiology, Neurosurgery and Neurology Loma Linda University School of Medicine Loma Linda CA USA

**Keywords:** hypoxia‐ischaemia, luciferase, miR‐17‐5p, neuroinflammation, NLRP3, PPAR‐β/δ, TXNIP

## Abstract

This study aimed to investigate the effects of PPAR‐β/δ receptor agonist GW0742 on neuroinflammation in a rat model of hypoxia‐ischaemia (HI) and in PC12 cells in OGD model. HI was induced by ligating the common carotid artery and inducing hypoxia for 150 minutes. Immunofluorescence was used for quantification of microglia activation and for determining cellular localization of PPAR‐β/δ. Expression of proteins was measured by Western blot. Activation of miR‐17‐5p by GW0742 was assessed in PC12 cells by Dual‐Luciferase Reporter Gene Assay. The endogenous expression of TXNIP, NLRP3, cleaved caspase‐1 and IL‐1β was increased after HI. GW0742 treatment significantly reduced the number of activated pro‐inflammatory microglia in ipsilateral hemisphere after HI. Mechanistically, GW0742 significantly decreased the expression of TXNIP, NLRP3, IL‐6 and TNF‐α. Either PPAR‐β/δ antagonist GSK3787, miR‐17‐5p inhibitor, or TXNIP CRISPR activation abolished the anti‐inflammatory effects of GW0742. Activation of PPAR‐β/δ by GW0742 activated miR‐17‐5p expression in PC12 cells and increased cell viability after OGD, which was accompanied by decreased expression of TXNIP and reduced secretion of IL‐1β and TNF‐α. In conclusion, GW0742 may be a promising neurotherapeutic for the management of HI patients.

## 1 INTRODUCTION

Hypoxic‐ischaemic encephalopathy (HIE) is a result of perinatal hypoxia‐ischaemia (HI), and it greatly contributes to child mortality and morbidity worldwide.^1^, ^2^, ^3^ Perinatal HI brain injury is often caused by disruption of placental blood flow and leads to impaired gas exchange, resulting in low oxygen and low metabolic substrates levels in the central nervous system. Cerebral palsy, epilepsy and learning disabilities are a potential long‐term neurological consequences of these detrimental events in the developing brain.^4^

It has been established that brain damage following HI is a complex disease with multiple contributing mechanisms and pathways. Neuroinflammation induced by activation of the resident and peripheral immune cells is increasingly recognized as a substantial pathogenic component of HI brain injury.^5^, ^6^ The NLRP3 inflammasome has been demonstrated to play a key role in detecting cellular damage and mediating inflammatory response to brain injury during ischaemia. NLRP3 inflammasome is a complex of NLRP3 (NACHT, LRR and PYD domains‐containing protein 3), apoptosis‐associated speck like (ASC) adapter protein and the downstream effector enzyme procaspase‐1. Activation of NLRP3 inflammasome initiates the cleavage of procaspase‐1 into the mature and active form of caspase‐1, which then converts the inactive pro‐IL‐1β into its active and secreted form. Pro‐inflammatory cytokines start or strengthen various signalling pro‐inflammatory pathways, which leads to cell death.^7^ Therefore, inhibition of pathways upstream of NLRP3 inflammasome can be a promising strategy to develop new therapies for stroke and HI.^8^

Thioredoxin‐interacting protein (TXNIP) is an upstream activator of NLRP3 induction.^9^ In response to reactive oxygen species (ROS), TXNIP binds to NLRP3 and starts inflammatory reaction.^10^ Our previous study has demonstrated that TXNIP was inhibited by PPAR‐β/δ receptor agonist GW0742—a process which was, at least partly, mediated through miR‐17‐5p.^11^ PPAR‐β/δ agonists have been shown to execute anti‐inflammatory effects in multiple disease models,^12^, ^13^, ^14^, ^15^ including CNS disease models.^16^ Pre‐treatment with GW0742 significantly inhibited inflammatory mediators in rats subjected to MCAO.^17^, ^18^ Our previous study showed that GW0742 reduced neuronal apoptosis, infarction volume and attenuated neurological deficits in rats in HI model. Here, we hypothesized that GW0742 has advantageous, anti‐inflammatory effects in NLRP3‐induced neuroinflammation after HI in the neonatal brain, which are regulated by PPAR‐β/δ, miR‐17‐5p and TXNIP (Figure S1). Furthermore, recent discoveries revealed that PPAR's can regulate the expression of certain miRNA's^19^, ^20^, ^21^ and PPAR response elements (PPRE) can be found in the promotor sequence of miR‐17‐5p. We therefore hypothesized that GW0742 can activate miR‐17‐5p transcription in PC12 cells, indicating that there is a regulatory mechanism controlling this pathway.

## 2 MATERIAL AND METHODS

### 2.1 Animals

All experimental protocols were approved by the Institutional Animal Care and Use Committee (IACUC) of Loma Linda University in accordance with the National Institute of Health (NIH) Guide for the Care and Use of Laboratory Animals. A total of 99 10‐day‐old unsexed Sprague Dawley rat pups (Envigo, Livermore, CA, USA) weighing 16‐20 g were used. Animals were housed in a controlled humidity and temperature room with a 12 hours light and dark cycle.

### 2.2 HIE Model

We used hypoxic‐ischaemic encephalopathy Rice‐Vannucci model, with some modification, as previously described.^22^ Briefly, 10‐day‐old neonatal rat pups were anesthetized with 3% isoflurane in air and maintained at 2% isoflurane in air. Temperature was controlled with heating blankets and incubators throughout the surgery and postoperatively. A small midline neck incision on the anterior neck was made, and the right common carotid artery (CCA) was isolated and gently separated from surrounding tissue. The carotid artery was ligated with 5‐O surgical suture, cut between the ligatures and skin was sutured. All surgeries were performed aseptically and lasted no longer than 8 minutes. After the surgery, the rats were allowed to recover for 1 hour. Thereafter, they were exposed to a hypoxic gas mixture of 8% oxygen and 92% nitrogen in an airtight jar kept at 37°C in the water bath. Hypoxia time was 150 minutes. For the sham animals, CCA was exposed without ligation or cutting, and pups were not subjected the hypoxia.

### 2.3 Drug administration

GW0742 (25 μg/kg; Tocris Bioscience) or vehicle was administered by intranasal method at 1 and 24 hours after HI. PPAR‐β/δ antagonist GSK3787 (300 μg/k; Abcam) or vehicle (1% DMSO in corn oil) was given by intranasal administration at 1 hour before HI and at 24 hours post‐HI. 0.5 nmol of LNA miR‐17‐5p inhibitor (antimiR, rno‐miR‐17‐5p miRCURY LNA miRNA Power Inhibitor; Exiqon) or control (miRCURY LNA miRNA Power Inhibitor Control; Exiqon) were injected intracerebroventricularly (ICV) during 3% isoflurane anaesthesia into the ipsilateral hemisphere at 24 hours before HI. 1 μg of TXNIP CRISPR activation plasmid (Santa Cruz Biotechnology, Dallas) or control CRISPR Activation Plasmid (Santa Cruz) were injected intracerebroventricularly to the ipsilateral hemisphere at 48 hours pre‐HI. Coordinates for ICV injection were as follows: 1.5 mm posterior, 1.5 mm lateral to the bregma and 2.5 mm deep into the ipsilateral hemisphere. Total volume of 2 μL of drug per pup was injected slowly in 5 minutes. The needle was left in place for an additional 10 minutes and then slowly withdrawn over 5 minutes to prevent backflow.

### 2.4 Western blotting

Western blot was performed as previously described.^23^ Proteins were loaded into SDS‐PAGE gel, and electrophoresis was performed. Then, the proteins were transferred to a nitrocellulose membrane, which was blocked with 5% non‐fat blocking grade milk (Bio‐Rad, Hercules) for 1 hour at room temperature. The membranes were incubated with the primary antibodies overnight at 4°C. The following primary antibodies were used: anti‐TXNIP (1:500; Santa Cruz Biotechnology), anti‐NLRP3 (1:1000; Novus Biologicals, Centennial), anti‐cleaved caspase‐1 (1:1000, Novus Biologicals), anti‐interleukin‐1β (1:1000; Santa Cruz Biotechnology), anti‐TNF‐α (1:500; Santa Cruz Biotechnology) and anti‐interleukin‐6 (1:1000; Santa Cruz Biotechnology). Anti‐β‐actin (1:3000; Santa Cruz Biotechnology) was used as loading control. The membranes were then incubated with appropriate secondary antibodies for 2 hours at room temperature. Immunoreactive bands were visualized with an ECL Plus kit (Amersham Biosciences) followed by exposure to X‐ray films and analysed using ImageJ software (NIH, Bethesda). For PC12 cells, expression of TXNIP and secreted pro‐inflammatory cytokines were measured from 4 separate experiments and normalized to expression of β‐actin in the cell lysate.^24^

### 2.5 Double immunofluorescence staining

Rat brain tissues were placed in formalin solution for 24 hours, embedded in OCT (Scigen Scientific, Paramount) and cut by cryostat. Brain sections were permeabilized with 0.3% Triton X‐100 for 30 minutes at room temperature and then blocked with 5% donkey serum at 37°C for 1 hour. Next, the sections were incubated at 4°C overnight with primary antibodies: anti‐PPAR‐β/δ (1:100; Abcam), anti‐Iba‐1 (1:400; Abcam), anti‐GFAP (1:400; Abcam), anti‐CD68 (1:100; Abcam) and anti‐iNOS (1:200; Abcam). Sections were washed in PBS, and respective fluorescence‐conjugated secondary antibodies were applied at the dilution of 1:200 for 1h at 37°C. Vectashield Antifade Mounting Medium with DAPI (Vector Laboratories, Burlingame) was used for mounting. Fluorescence microscope (Leica DMi8, Leica Microsystems) was used to capture images.

### 2.6 Microglia quantification

Each stained section was processed under identical gain and laser power setting. Four different sections from each rat were stained and analysed. Activation of microglia was evaluated on the basis of cellular morphology and Iba‐1 intensity exceeding the set threshold, and additionally by quantifying CD68 positive cells.^25^ Average microglia soma size was calculated using ImageJ software. Activation of pro‐inflammatory microglia was evaluated by double positive CD68/iNOS‐stained cells quantification. Images were acquired by use of fluorescence microscope (Leica DMi8; Leica Microsystems) and analysed with the use of ImageJ software.

### 2.7 Cell culture

PC12‐rat pheochromocytoma cells (ATCC, American Type Culture Collection, Manassas) were grown as previously described.^26^ Cells were cultured in the incubator at 37°C and 5% CO_2_ in F12‐K medium supplemented with 2.5% (v/v) foetal bovine serum (FBS), 15% (v/v) horse serum (HS) and 1% (v/v) penicillin‐streptomycin solution (full growth complete medium). Growing media was replaced every 3 days until the cells reached 70% confluence. PC12 cells were used at passage 7‐10. For Western blotting, cells were plated at a density of 25 000 cells/well in a 6‐well plate. For experiments performed in 96‐well plates, cells were plated at 10 000 cells/well. In all experiments, an equal amount of the vehicle (0.1% DMSO final concentration) was used as control.

### 2.8 Oxygen glucose deprivation model

Full growth complete medium was replaced with no‐glucose medium (pure FK12 medium without the addition of horse serum, FBS or antibiotics), and cells were placed in a hypoxic chamber and flushed with 1% oxygen for 3 hours. Complete growth media were then added, and cells were allowed to recover for 24 hours, after which the cells were prepared for cell viability assay and Western blotting.^26^

### 2.9 Cell viability assay

Cell viability was measured using Cell Counting Kit‐8 (CCK‐8) (Dojindo).^27^ PC12 cells were seeded in 96‐well plates at 10 000 cells/well and were treated with 0, 0.1, 1, 10 and 100 μmol/L of GW0742 or vehicle (0.1% DMSO) for 2 hours before OGD or/and for 24 hours immediately after OGD. CCK‐8 solution was added to the culture medium at 1/10 volume and incubated at 37°C for 2 hours. After that, the absorbance was measured at 450 nm using Micro‐plate reader (SpectraMax^®^ i3x; Molecular devices, San Jose). Cell viability was normalized to the average absorbance of control group.

### 2.10 Plasmid transfection

Construct plasmids: pGL3‐miR‐17‐5p‐Sensor and pGL3‐miR‐17‐5p‐Control were purchased from Addgene and were a gift from Joshua Mendell (Addgene plasmid # RRID:Addgene_21166 and RRID:Addgene_21167). Sensor and control luciferase constructs which we used were made by ligating oligonucleotides containing two sites with perfect complementarity to miR‐17‐5p into the XbaI site of the pGL3‐control vector (Promega, Madison).^28^ Prior to transfection, differentiated PC12 cells were allowed to reach 70% confluence in 96‐well plates. Briefly, plasmid solutions were mixed with 125 µL Opti‐MEM. In a separate tube, 2 µL of Lipofectamine 2000 (Invitrogen, Carlsbad) was mixed with 125 µL of Opti‐MEM. The two tubes were combined and left to sit at room temperature for 10 minutes. 10 µL mixture/well was applied to cells grown in complete media. Then, the cells were placed in the incubator, and after 24 hours, the Luciferase Reporter Gene Assay was performed.

### 2.11 Luciferase reporter gene assay

Twent‐four hours before transfection, PC12 cells were plated at 10 000 cells per well in a 96‐well plate and incubated in complete growth medium. 100 ng of sensor or control plasmid together with 40 ng of pRL‐SV40 (Promega) were transfected in combination with vehicle (DMSO) or 1 μmol/L or 10 μmol/L of GW0742, using Lipofectamine 2000 (Invitrogen). Luciferase assays were performed 24 hours after transfection. Luciferase activity was measured using the Dual‐Glo Luciferase Assay System (Promega) with a luminometer (SpectraMax^®^ i3x; Molecular devices). For each transfected well firefly luciferase activity was normalized to Renilla luciferase activity. Independently prepared plasmids were transfected three times on different days, with the luciferase assays conducted each time. Each transfected well was assayed in triplicate.^28^

### 2.12 Statistical analysis

Statistical analyses were performed with GraphPad Prism 6. The data were expressed as means ± SD. Differences between groups were first compared using analysis of variance (one‐way ANOVA), and then, post hoc testing was conducted with Tukey, Sidak or Dunnett's multiple comparisons. Differences between two groups were compared using Student's *t* test. All reported *P* values were two‐sided, and a value of *P* < 0.05 was considered statistically significant.

## 3 RESULTS

### 3.1 TXNIP and NLRP3 inflammasome pathway are activated after HI

**FIGURE 1:**
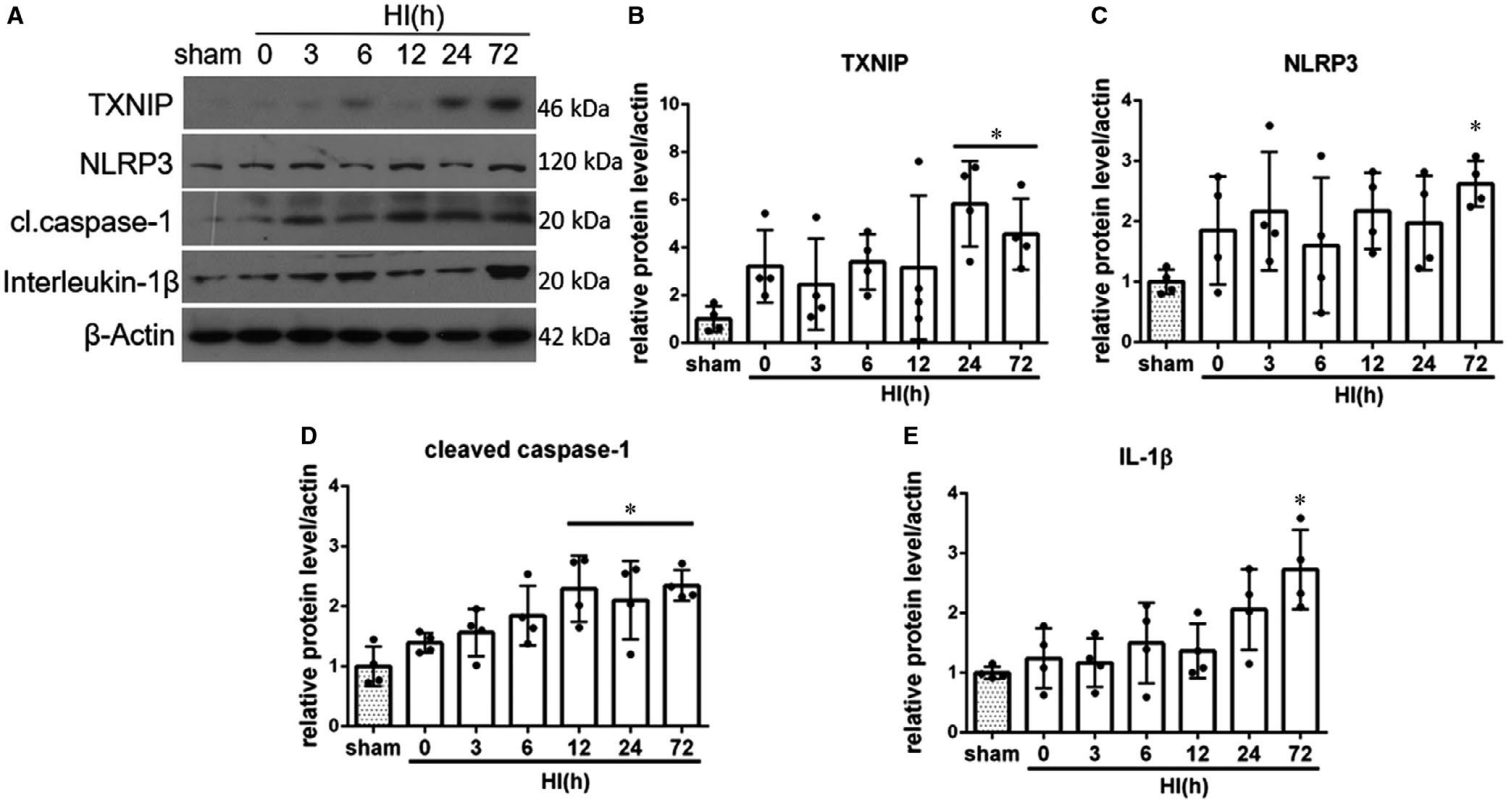
Temporal expression of endogenous TXNIP, NLRP3, cleaved caspase‐1 and interleukin‐1β in the ipsilateral brain hemisphere after hypoxia‐ischemia (HI). A, Representative pictures of Western blot data. B, Western blot data analysis showed that endogenous TXNIP expression levels significantly increased at 24 and 72 h post‐HI. C, Western blot data showed that endogenous NLRP3 expression levels increased, reaching significance at 72 h after HI. D, Cleaved caspase‐1 expression was significantly increased at 12, 24 and 72 h post‐HI. E, Active interleukin‐1β expression was increased after HI, reaching statistical significance at 72 h after HI. *P < 0.05 vs sham. Data are represented as mean ± SD, n = 4 for each group

As shown in Figure 1A,B, the expression of TXNIP was up‐regulated after HI compared to sham and reached significance at 24 and 72 hours after HI (*P*  <  0.05). NLRP3 expression was significantly higher compared with sham group and peaked at 72 hours (Figure 1C, *P*  <  0.05). The level of endogenous cleaved caspase‐1 was significantly increased at 12, 24 and 72 hours after HI (Figure 1D, *P*  <  0.05). The data showed that expression of interleukin‐1β was increased after HI, reaching significance at 72 hours post‐HI (Figure 1E, *P * <  0.05, Figure S2). Overall these results indicate that inflammasome pathway is activated after HI.

### 3.2 GW0742 activated PPAR‐β/δ in microglia at 72 hours after HI

**FIGURE 2:**
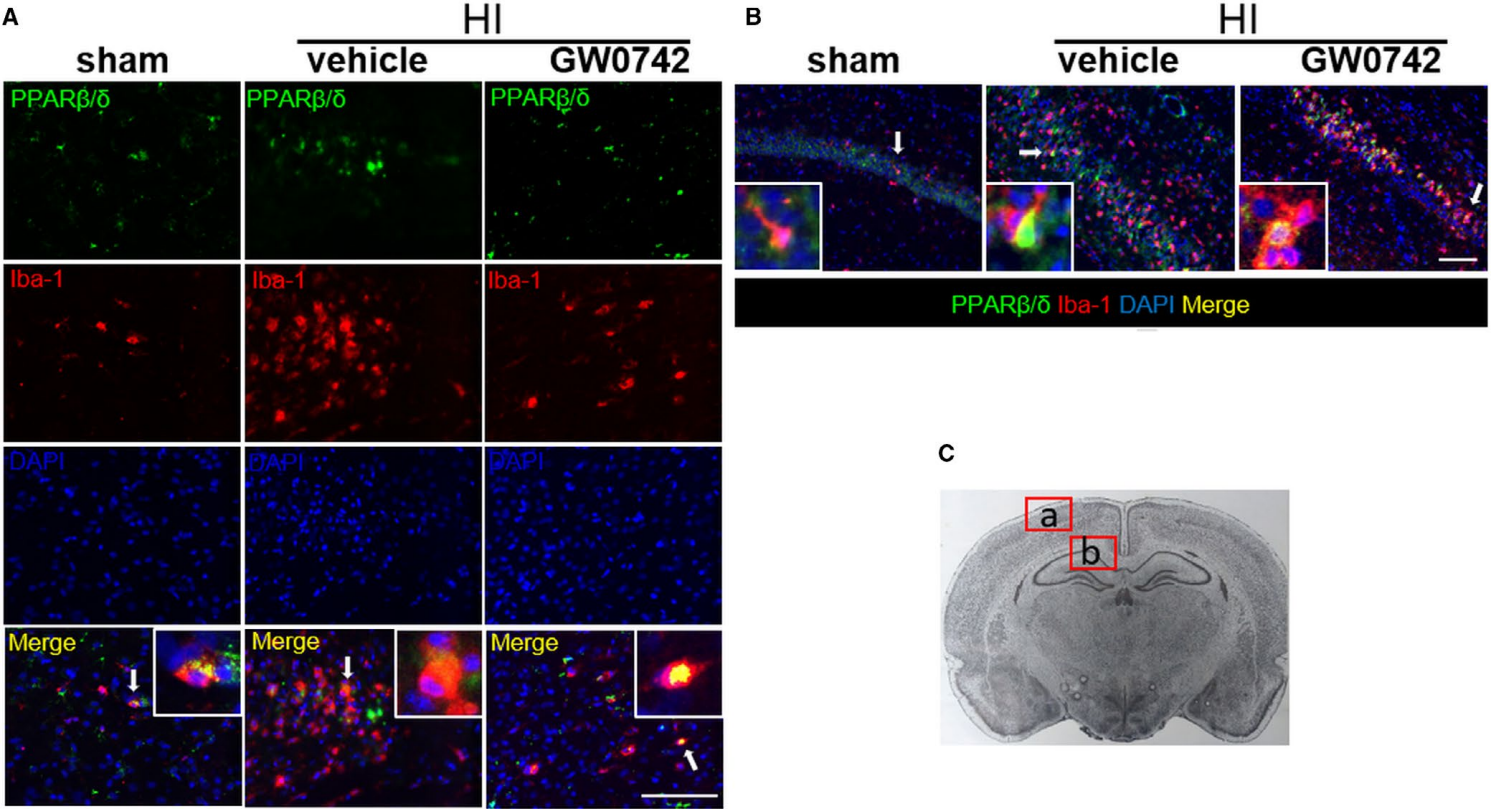
PPAR‐β/δ is expressed in microglia in neonatal rat brain. A, Representative immunofluorescence staining of PPAR‐β/δ and the microglia marker Iba‐1 in the cerebral cortex at 72 h post‐HI. B, Representative immunofluorescence staining of PPAR‐β/δ and the microglia marker Iba‐1 in the hippocampus at 72 h post‐HI. PPAR‐β/δ was colocalized with Iba‐1 in the sham, vehicle and GW0742 treatment (25 μg/kg) group. Compared with the sham and vehicle group, there was higher nuclear expression of PPAR‐β/δ in microglia in GW0742 treated group. C, Panel indicates the location of staining showed in (A, B). Picture taken from: *Paxinos and Watson. The rat brain in stereotaxic coordinates*. Green is for PPAR‐β/δ, red is for Iba‐1, and blue is for DAPI. Arrows indicate cells, which are shown in higher magnification in the insets. 2 rats for each group. Scale bar‐ 100 μm

Double immunofluorescent staining was conducted to colocalize PPAR‐β/δ with microglia. As shown in Figure 2A, PPAR‐β/δ showed to be colocalized with Iba‐1, a microglial marker, in the cerebral cortex in sham rats and at 72 hours after HI. PPAR‐β/δ was also found to be expressed in microglia in the hippocampus—colocalization of PPAR‐β/δ with microglia in CA1 region of the hippocampus of sham, HI + vehicle and HI + GW0742‐treated rats is shown in Figure 2B. Intensified nuclear expression of PPAR‐β/δ was observed in GW0742 treated rats, suggesting PPAR‐β/δ activation.

### 3.3 GW0742 enhanced PPAR‐β/δ in astrocytes at 72 hours after HI

**FIGURE 3:**
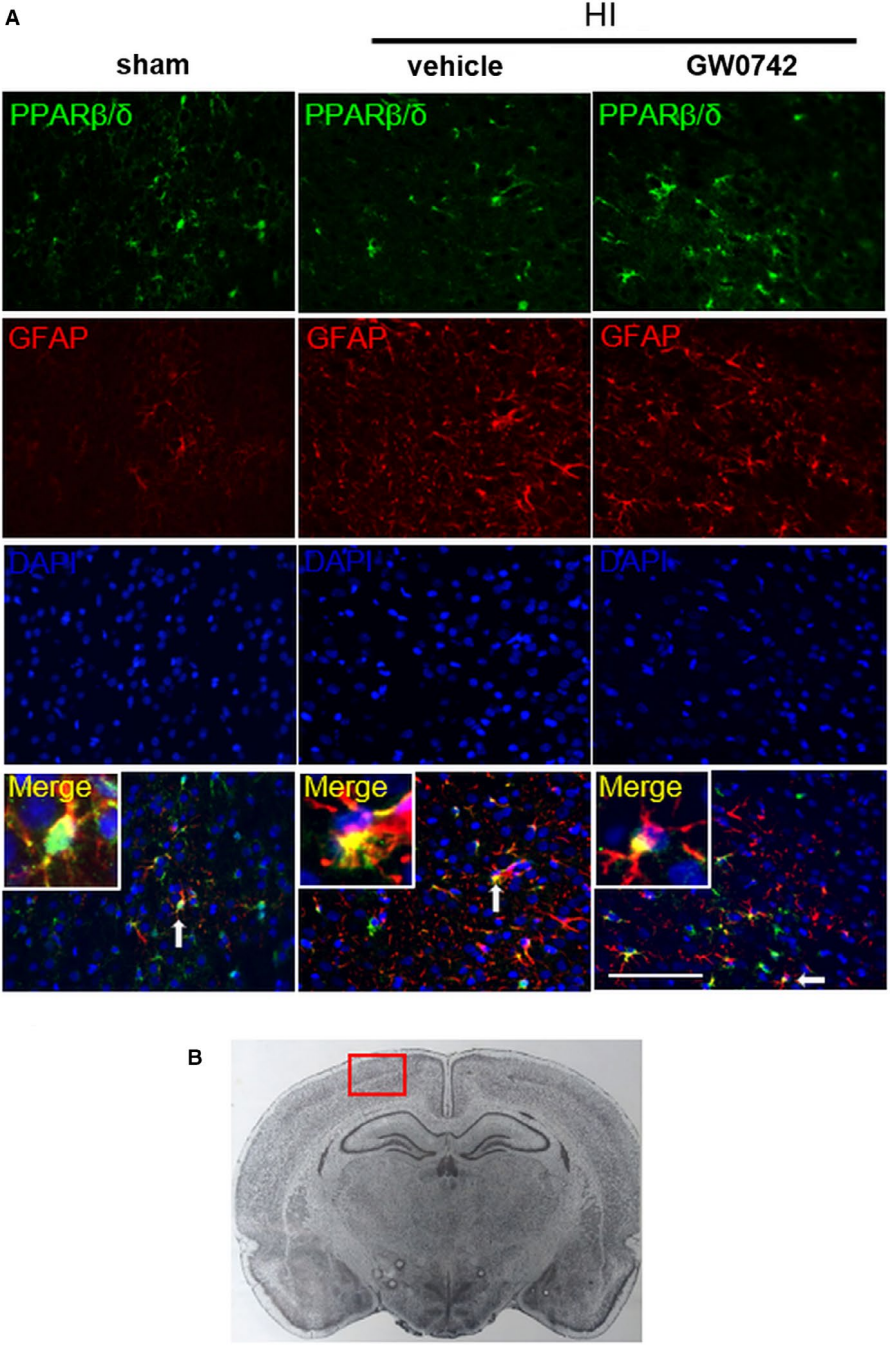
PPAR‐β/δ is expressed in astrocytes in neonatal rat brain. A, Representative immunofluorescence staining of PPAR‐β/δ and the astrocyte marker GFAP in the cerebral cortex at 72 h post‐HI. PPAR‐β/δ was colocalized with GFAP in the sham, vehicle and GW0742 treatment (25 μg/kg) group. Compared with the sham and vehicle group, there was higher expression of PPAR‐β/δ in astrocytes in GW0742 treated group. B, Panel indicates the location of staining showed in (A). Green is for PPAR‐β/δ, red is for GFAP, and blue is for DAPI. Arrows indicate cells, which are shown in higher magnification in the insets. 2 rats for each group. Scale bar‐100 μm

We also found that PPAR‐β/δ was expressed in astrocytes at 72 hours after HI. Colocalization of astrocytic marker GFAP with PPAR‐β/δ in cerebral cortex of rats from sham, HI + vehicle and HI + GW0742 groups is shown in Figure 3A. Enhanced expression of GFAP after HI was observed showing activation of astrocytes.

### 3.4 GW0742 treatment suppressed pro‐inflammatory microglia activation in ipsilateral hemisphere at 72 hours after HI

**FIGURE 4:**
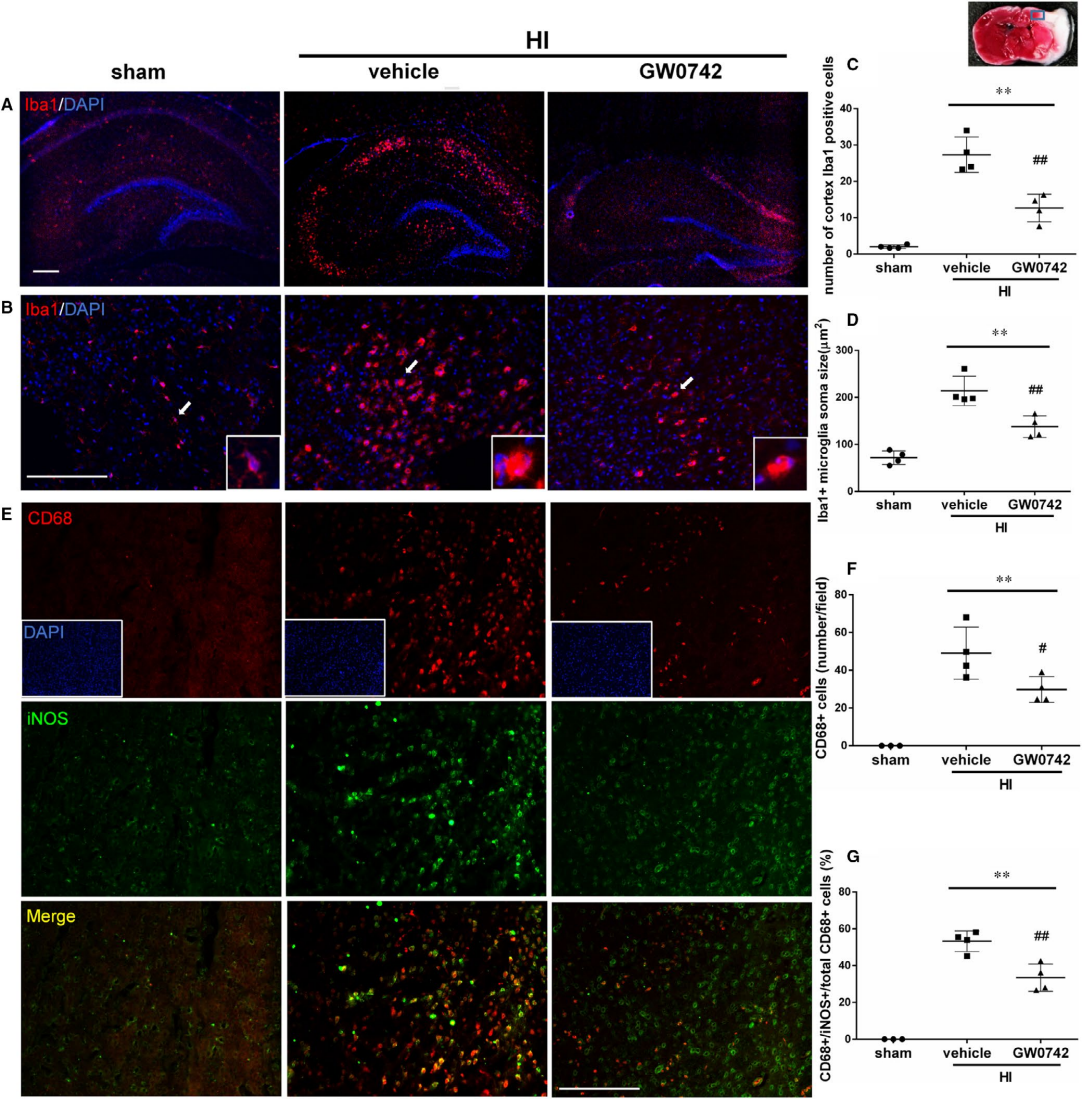
GW0742 suppresses pro‐inflammatory microglia activation at 72 h after HI. A, Representative images of Iba‐1‐positive microglia in the hippocampus of ipsilateral hemisphere of sham, vehicle‐treated and GW0742‐treated HI rats. Red is for Iba‐1 and DAPI (blue) stains for cell nucleus. B, Representative images of Iba‐1‐positive microglia in cerebral cortex of ipsilateral hemisphere of sham, vehicle‐treated and GW0742‐treated HI rats. Arrows indicate cells, which are shown in higher magnification in the insets. C, Quantitative cell counts of Iba‐1‐ positive microglia and (D) measurements of microglia soma size in the ipsilateral cortex of sham, vehicle and GW0742‐treated HI rats. n = 4. E, Representative images of CD68 (for activated microglia) and iNOS (proinflammatory microglia) stained microglia in the cerebral cortex of ipsilateral hemisphere of sham, vehicle‐treated and GW0742‐treated HI rats. F, Quantitative cell counts of CD68‐positive activated microglia and G, double positive CD68/iNOS + cells percentage of pro‐inflammatory microglia. n = 4. Data are presented as the mean ± SD. **P < 0.01 vs sham, ^#^ *P* < 0.05 vs HI + vehicle, ^##^ *P* < 0.01 vs HI + vehicle. ANOVA with post hoc Tukey multiple comparison test. Scale bar‐ 200 μm

In response to hypoxic‐ischaemic injury, activated microglia are the major source of inflammatory cytokines and chemokines which cause cytotoxic effects.^29^, ^30^ To explore whether GW0742 can affect the microglial response after HI, we first used Iba‐1 as microglial marker in the peri‐infarct area at 72 hours after HI. Iba‐1 expression in the hippocampus (Figure 4A) and cortex (Figure 4B) in ipsilateral hemispheres were increased after HI. The number of microglia in cerebral cortex significantly increased in vehicle and GW0742‐ treated rats when compared to sham group (Figure 4C, *P* < 0.01). Furthermore, microglia in vehicle and GW0742‐treated animals demonstrated bigger soma with shorter cell processes than sham animals (Figure 4D, *P* < 0.01). Quantitative analysis of microglia activation revealed a significant reduction in Iba‐1 + cell numbers (Figure 4C, *P* < 0.01), as well as decreased soma size (Figure 4D, *P* < 0.01) in GW0742‐treated rats in ipsilateral hemisphere compared to vehicle group. To further validate our findings, we stained brain sections with a marker of activated microglia CD68 together with iNOS, which is expressed by pro‐inflammatory microglia.^31^ Colocalization of CD68 and iNOS is indicative of a pro‐inflammatory microglia phenotype. We found that rats from vehicle and GW0742‐treated group displayed higher numbers of CD68‐positive activated microglia compared to sham group (Figure 4E,F, *P* < 0.01). Treatment with GW0742 resulted in a reduced number of activated microglia compared to vehicle group (Figure 4E,F, *P* < 0.05). Moreover, GW0742 suppressed the pro‐inflammatory microglia activation, which was manifested by lower percentage of CD68/iNOS double positive cells in the ipsilateral hemisphere compared to vehicle group (Figure 4G, *P* < 0.01).

### 3.5 GW0742 suppressed HI‐induced NLRP3 activation and neuroinflammation, which was abolished by either GSK3787, ANTIMIR‐17‐5p or TXNIP CRISPR activation at 72 hours after HI

In our previous study, we showed that TXNIP expression was significantly increased in HI + vehicle group when compared with sham (*P* < 0.05) at 72 hours after HI, and GW0742 treatment significantly decreased the expression of TXNIP compared with vehicle group (*P* < 0.05).^11^ Furthermore, we demonstrated that blocking PPAR‐β/δ with GSK3787 and inhibition of miR‐17‐5p significantly increased TXNIP expression. Additionally, we validated TXNIP CRISPR activation.^11^

**FIGURE 5:**
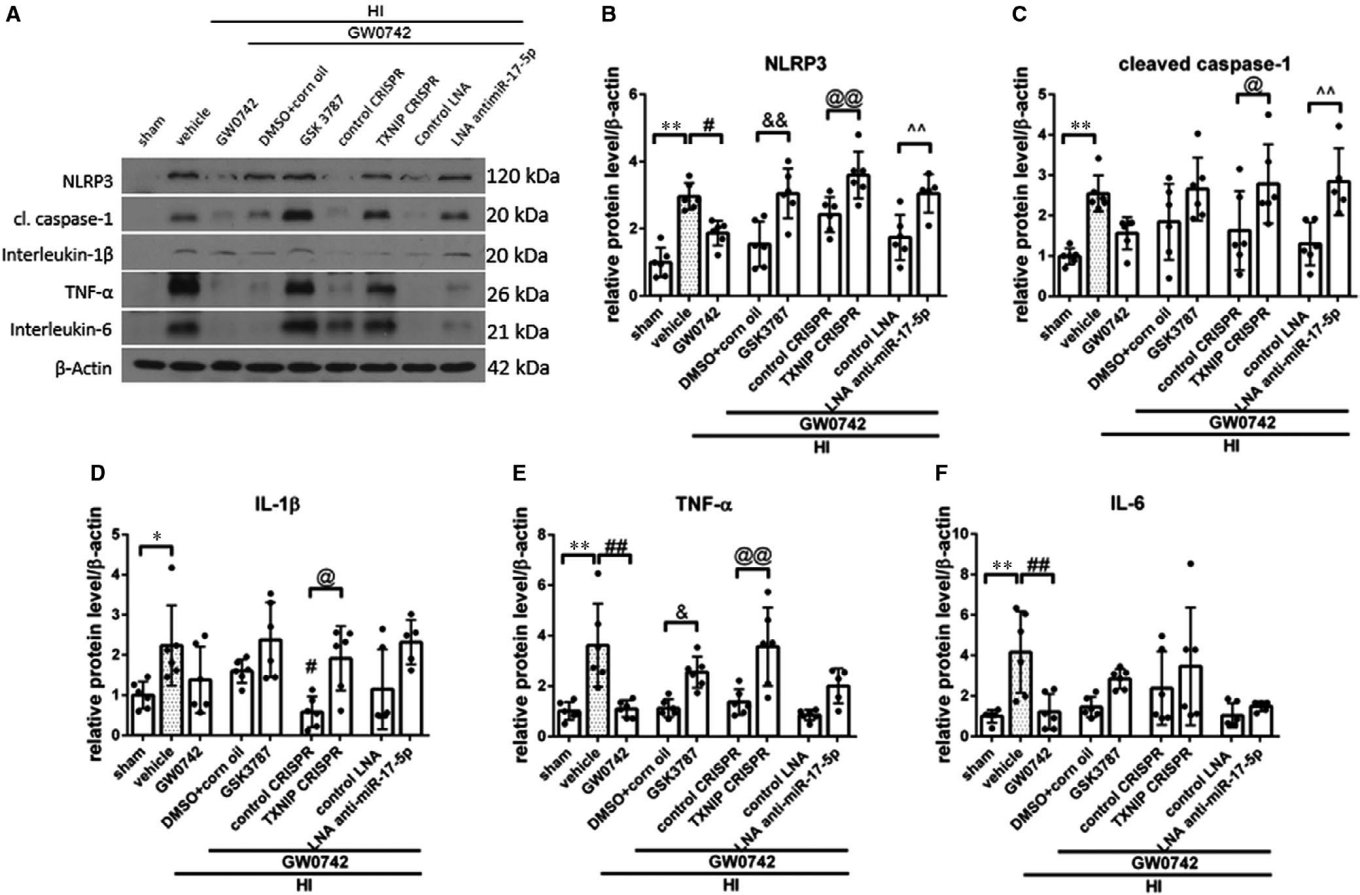
Effects of GW0742 on neuroinflammation via the PPAR‐β/δ/miR‐17‐5p/TXNIP/NLRP3 signalling pathway at 72 h post‐hypoxia‐ischemia (HI). A, Representative picture of Western blot data showing bands of the expression levels of NLRP3, cleaved caspase‐1, interleukin‐1β, TNF‐α and interleukin‐6 either with GW0742 treatment alone or GW0742 + DMSO in corn oil, GW0742 + GSK3787, GW0742 + control CRISPR, GW0742 + TXNIP CRISPR, GW0742 + control LNA antimir, GW0742 + LNA antimir‐17‐5p groups. B‐F, Western blot data quantification of bands showed that GW0742 significantly decreased NLRP3, TNF‐α and interleukin‐6 compared with HI + vehicle. GSK3787 showed to significantly increase NLRP3 and TNF‐α expression when compared with GW0742 + DMSO in corn oil. TXNIP CRSIPR activation showed to significantly increase NLRP3, cleaved caspase‐1, interleukin‐1β and TNF‐α compared with GW0742 + control CRISPR group. LNA antimir‐17‐5p showed to significantly increase NLRP3 and cleaved caspase‐1 when compared to GW0742 + control LNA group. *P < 0.05, sham vs vehicle; **P < 0.01, sham vs vehicle; ^#^ *P* < 0.05, HI + vehicle vs HI + GW0742; ^##^ *P* < 0.01, HI + vehicle vs HI + GW0742; ^&^ *P* < 0.05, HI + GW0742+GSK3787 vs HI + GW0742+DMSO + corn oil; ^&&^ *P* < 0.01, HI + GW0742+GSK3787 vs HI + GW0742+DMSO + corn oil; ^@^ *P* < 0.05, HI + GW0742+TXNIP CRISPR vs HI + GW0742+ control CRISPR; ^@@^ *P* < 0.01, HI + GW0742+TXNIP CRISPR vs HI + GW0742+ control CRISPR; ^^^^ *P* < 0.01, HI + GW0742+LNA antimir‐17‐5p vs HI + GW0742+ control LNA. n = 6 for each group. n = 5 for HI + GW0742+LNA antimir‐17‐5p. ANOVA with post hoc Sidak comparison test

Western blot data revealed that NLRP3 (*P* < 0.01), cleaved caspase‐1 (*P* < 0.01), interleukin‐1β (*P* < 0.05), TNF‐α (*P* < 0.01) and interleukin‐6 (*P* < 0.01) expression significantly increased in vehicle group when compared with sham at 72 hours after HI (Figure 5A‐F, Figure S3). GW0742 treatment significantly decreased NLRP3 (Figure 5B, *P* < 0.05), TNF‐α and inerleukin‐6 expression when compared with HI + vehicle (Figure 5E,F, *P* < 0.01). Expressions of cleaved caspase‐1 and interleukin‐1β were also decreased; however, they did not reach statistical significance (Figure 5C,D). Rats were treated with GSK3787, TXNIP CRISPR activation plasmid and LNA antimiR‐17‐5p to investigate the mechanism of GW0742‐induced suppressing effects on neuroinflammation. GSK3787 significantly increased NLRP3 expression (Figure 5A,B, *P* < 0.01), therefore reversing the effect of GW0742 and leading to significant increase of expression of TNF‐α, compared to respective DMSO + corn oil control group (Figure 5E, *P* < 0.05). Moreover, direct activation of TXNIP suppressed the GW0742 induced inhibition of NLRP3/cleaved caspase‐1 pathway compared to control group (Figure 5B,C, *P* < 0.01 and *P* < 0.05, respectively), which was accompanied by significantly increased expression of interleukin‐1β (Figure 5D, *P* < 0.05) and TNF‐α (Figure 5E, *P* < 0.01). Inhibition of miR‐17‐5p was associated with significantly increased NLRP3 and cleaved caspase‐1 expression compared to control LNA group (Figure 5B,C, *P* < 0.01), which was an indication of inflammasome activation. These changes were accompanied by increased expression of interleukin‐1β, TNF‐α and interleukin‐6 in the ipsilateral hemisphere compared to control LNA group (Figure 5D‐F); however, they were not statistically significant. In our previous study, we have validated miR‐17‐5p inhibition—injection of miR‐17‐5p inhibitor resulted in dramatic decrease of miR‐17‐5p level in neonatal rat brain.^11^

### 3.6 GW0742 inhibited cell death, blocked TXNIP expression and reduced inflammation after OGD in PC12 cells in vitro

**FIGURE 6:**
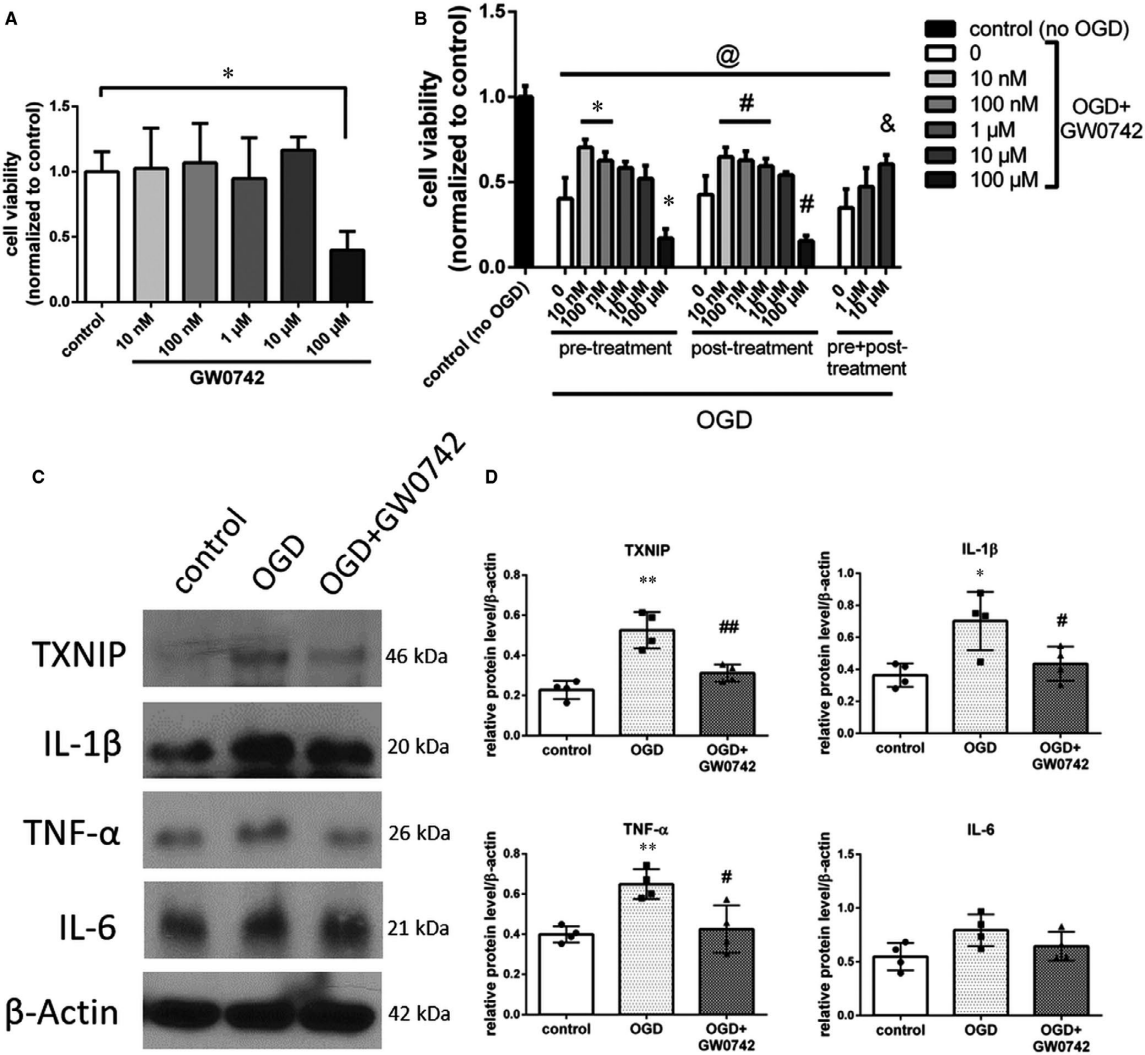
GW0742 protected PC12 cells and reduced the OGD‐induced increase in the expression levels of pro‐inflammatory mediators in vitro. A, Cell viability quantification of PC12 cells, which were exposed to vehicle or 10 nmol/L, 100 nmol/L, 1 μmol/L, 10 μmol/L and 100 μmol/L of GW0742 for 24 h. *P < 0.05 vs control. ANOVA followed with post hoc Dunnett's test. B, Cell viability quantification of PC12 cells, which were exposed to 3h of OGD and vehicle or 10 nmol/L, 100 nmol/L, 1 μmol/L, 10 μmol/L and 100 μmol/L of GW0742 in 2 h pre‐treatment, 24 h post‐treatment or combined experimental regimens. ^@^ *P* < 0.05 vs no OGD control, *P < 0.05 vs pre‐treatment control, ^#^ *P* < 0.05 vs post‐treatment control, ^&^ *P* < 0.05 vs pre + post‐treatment control. ANOVA followed with post hoc Tukey's test. C, Representative pictures of expression levels of TXNIP, and secreted interleukin‐1β, TNF‐α and interleukin‐6 in control, OGD + vehicle and OGD + GW0742‐treated PC12 cells analysed by Western blot. D, Quantification of proteins expression. OGD significantly increased the expression of TXNIP in PC12 cells and increased expression of secreted interleukin‐1β and TNF‐α. Pre‐ and post‐treatment with GW0742 significantly decreased expression of TXNIP, interleukin‐1β and TNF‐α at 24 h post‐OGD. Results are presented as mean ± SD. *P < 0.05 vs control, **P < 0.01 vs control, ^#^ *P* < 0.05 vs OGD, ^##^ *P* < 0.01 vs OGD. Analysed by ANOVA followed by Tukey's post hoc test. n = 4 per group

Previously, it was revealed that incubation with various doses of GW0742 resulted in neuroprotective biological effects in vitro^32^, ^33^, ^34^, ^35^, ^36^, ^37^; however, the effect of GW0742 on PC12 cells has not been investigated. We exposed PC12 cells to GW0742 concentrations in a range of 10 nmol/L to 100 µmol/L for 24 hours to determine toxicity, and then, the CCK‐8 assay was performed. GW0742 doses ranging from 10 nmol/L to 10 µmol/L did not affect the cell viability. However, the survival of PC12 cells significantly decreased when they were incubated with 100 µmol/L of GW0742 (Figure 6A, *P* < 0.05). Similar neurotoxic effects of GW0742 at 100 µmol/L were reported before in cerebellar neurons.^37^ Then, we exposed PC12 cells to 3 hours of OGD conditions, which resulted in significant cell death compared to control group (Figure 6B, *P* < 0.05). We tested 0.01‐100 µmol/L concentrations of GW0742 in 3 treatment regimens: pre‐treatment starting at 2 hours before OGD, post‐treatment for 24 hours after OGD, and combined approach with pre‐ and post‐treatment. Pre‐treatment with 10 and 100 nmol/L significantly increased cell viability compared to control, while 100 µmol/L significantly decreased cell viability after OGD (Figure 6B, *P* < 0.05). Consistently, post‐OGD treatment with 10 nmol/L, 100 nmol/L and 1 µmol/L significantly increased cell viability compared to control, while 100 µmol/L significantly decreased cell viability after OGD (Figure 6B, *P* < 0.05). Treatment with 10 µmol/L in both treatment regimens tended to increase cell viability; however, it was not statistically significant. There was also no significant difference between 10 nmol/L, 100 nmol/L, 1 µmol/L and 10 µmol/L treatment groups (*P* > 0.05). Finally, we subjected the cells to OGD and both treatment regimens combined. We found that pre‐ and post‐treatment with 10 µmol/L significantly increased cell viability at 24 hours after OGD (Figure 6B, *P* < 0.05). We chose the combined pre‐ and post‐treatment with 10 µmol/L of GW0742 as optimal treatment regimen for mechanism analysis by Western blot.

We found that OGD significantly increased the expression of TXNIP (*P* < 0.01), IL‐1β (*P* < 0.05) and TNF‐α (*P* < 0.01) compared to control group (Figure 6C,D, Figure S4). Treatment with 10 µmol/L of GW0742 significantly reduced expression of TXNIP (Figure 6C,D, *P* < 0.01), which was accompanied by decreased expression of secreted pro‐inflammatory cytokines IL‐1β and TNF‐α (Figure 6C,D, *P* < 0.05), suggesting that GW0742 can reduce inflammation in PC12 cells subjected to OGD.

### 3.7 GW0742 regulated MIR‐17‐5p expression in PC12 cells

**FIGURE 7:**
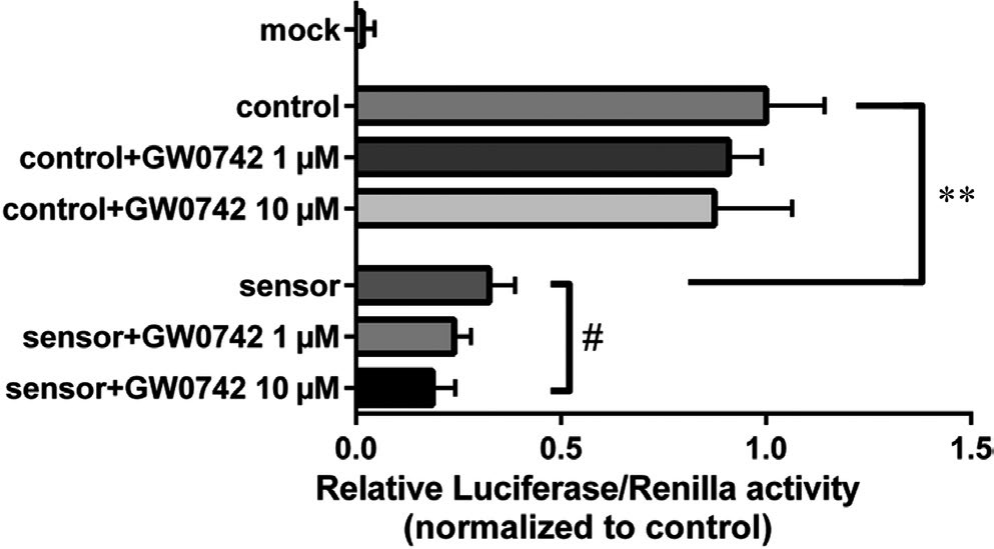
GW0742 regulates miR‐17‐5p expression. Control and miR‐17‐5p sensing constructs were transfected to PC12 cells. Cells were then treated with vehicle or 1 or 10 μmol/L of GW0742 for 24 h. Co‐transfection of sensor plasmids with 10 μmol/L of GW0742 significantly decreased expression of luciferase constructs compared to vehicle‐treated sensor group, indicating activation of miR‐17‐5p by GW0742. Data presented as means ± SD, **P < 0.01, control vs sensor; ^#^ *P* < 0.05, sensor vs sensor + 10 μmol/L GW0742. N = 3

To check if PPAR‐β/δ can regulate miR‐17‐5p, we used PC12 cells and treated them with GW0742, which has been shown to selectively activate PPAR‐β/δ.^38^ To measure miR‐17‐5p activation, PC12 cells were transfected with constructs expressing Luciferase. Sensor pGL3 luciferase reporter construct with sites perfectly complementary to miR‐17‐5p in the 3′‐untranslated region (UTR) which binds miR‐17‐5p, and control constructs with the reverse‐complementary sequence of the miRNA‐binding site which don't bind miR‐17‐5p were used.^28^ MicroRNA binds to 3′UTR of Luciferase sensor leading to blockage of Luciferase expression. Therefore, the decrease of Luciferase activity was an indication of miR‐17‐5p expression activation. When PC12 cells were incubated with sensor constructs, there was an ~65% reduction in luciferase activity compared with control constructs (Figure 7, *P* < 0.01). This significant inhibition of the luciferase signal demonstrated that PC12 cells endogenously express miR‐17‐5p. Co‐transfection of control plasmids with 1 μmol/L or 10 μmol/L of GW0742 did not significantly change the expression of Luciferase (Figure 7). Meanwhile, co‐transfection of sensor plasmids with 10 μmol/L of GW0742 significantly decreased (by ~42%) expression of luciferase constructs compared to control sensor group (Figure 7, *P* < 0.05), indicating activation of miR‐17‐5p by GW0742.

## 4 DISCUSSION

Elucidation of the inflammatory mechanisms involved in neonatal hypoxic‐ischaemic injury can help to find therapeutic strategies for prevention and treatment of neurological handicaps in maturing infants.^39^ Our study demonstrates that targeting inflammatory pathways by activating PPAR‐β/δ receptor with GW0742 can be a potential treatment for HI. Anti‐inflammatory effects of GW0742 were mediated through TXNIP, PPAR‐β/δ and miR‐17‐5p. Furthermore, we demonstrated the novel mechanism of regulation of miR‐17‐5p by PPAR‐β/δ nuclear receptor.

We found that TXNIP and NLRP3 expression is increased after HI. It was previously shown that after HI there was a robust amount of NLRP3 protein pulled down together with TXNIP compared with sham animals, indicating that TXNIP binds to NLRP3 and activates the inflammasome.^40^ Other studies identified increased expression of TXNIP and NLRP3 at 24h after the ischaemic insult.^9^, ^41^, ^42^ Here, we showed that TXNIP and NLRP3 expression is induced even at 72 hours after HI, which is accompanied by caspase‐1 cleavage. Consistently with other studies demonstrating that neonatal HI results in a sustained increase in expression of IL‐1β mRNA and protein^43^, ^44^, ^45^ in the damaged ipsilateral hemisphere, we found increase in protein expression of IL‐1β in the ipsilateral hemisphere, which was significant at 72 hours after HI. Our results, together with existing literature, show that NLRP3 inflammasome is activated after HI, which contributes to neuroinflammation in neonatal brain.

The anti‐inflammatory actions of PPAR‐β/δ agonists have been reported in a various cell types, including astrocytes and microglia.^12^, ^16^ In our study, PPAR‐β/δ was expressed in the neonatal rat brain in astrocytes and microglia after HI and after GW0742 treatment, which suggests that those cells are involved in a protective mechanism induced by PPAR‐β/δ activation. We showed that activation of PPAR‐β/δ by GW0742 reduced inflammation in ipsilateral hemispheres of HI rats and in neuronal PC12 cells after OGD. PPAR‐β/δ can control inflammation by activating gene transcription by directly binding to PPRE in the promotor region of target genes, or independently of binding to PPREs, by mechanism called trans‐repression, where PPAR ligand antagonizes the actions of other transcription factors, like nuclear factor‐κB (NF‐κB). Trans‐repression is thought to underlie many of the anti‐inflammatory effects of PPAR‐β/δ.^46^, ^47^, ^48^, ^49^ The consensus is that the one of the major pathogenic factors in the perinatal HI brain injury is inflammation induced by the activation of microglia and infiltrating macrophages. The inflammatory response to HI is driven primarily by microglial cells producing pro‐inflammatory factors, that is cytokines, chemokines and nitric oxide synthase (NOS), which are contributing to neurological disorders.^30^ Under physiologic conditions microglia is in a ramified, resting state, but in reaction to the HI injury resting microglia cells proliferate and progress into reactive cells in a process called microglial activation.^29^ During activation, microglial cells become enlarged with short thick processes and can migrate to the site of injury, where they work in cooperation with recruited monocytes/macrophages to activate an immune response.^50^ We found reduced number of activated microglia in the hippocampus as well as the cerebral cortex in the ipsilateral hemispheres after GW0742 treatment. The microglial cells in the GW0742‐treated brains were smaller, they were less numerous, with more ramified morphology, and they expressed lower levels of iNOS. These changes were accompanied by decreased expression of pro‐inflammatory cytokines in ipsilateral hemispheres after HI. Additionally, we found similar protective anti‐inflammatory effect in PC12 cells in OGD model after GW0742 treatment. Our results add another piece of evidence to highlight an important role of PPAR‐β/δ in silencing the pathogenic activation of inflammation.

TXNIP is an oxidative stress‐sensitive, upstream activator ^51^ which can bind to and activate NLRP3 inflammasome.^52^, ^53^ We found that activation of PPAR‐β/δ resulted in suppression of TXNIP/NLRP3 pathway. Previous research demonstrated that knockout or pharmacological inhibition of TXNIP resulted in restoration of redox balance, inhibition of TXNIP‐NLRP3 pathway, and consequently reduced brain infarction rate and improved neurological scores in embolic stroke model in mice.^54^ Another study indicated that curcumin protected mouse hippocampi from glutamate toxicity by blocking TXNIP/NLRP3 inflammasome activation and endoplasmic reticulum stress.^55^ Furthermore, it was showed that Umbelliferone limited TXNIP/NLRP3 pathway activation which was accompanied by the reduction of inflammatory cytokines level.^9^ Our results, together with aforementioned evidence, point to TXNIP as an important hub connecting various stressors with inflammation and identify TXNIP as a potential therapeutic target in brain injury‐related neuroinflammation.

TXNIP is a validated miR‐17‐5p target.^56^ It was shown that miR‐17‐5p blocks TXNIP in rat pancreatic β‐cells^57^ and miR‐17 down‐regulation stabilized TXNIP in mouse neural stem cells.^58^ Previously, we found that GW0742 treatment increased the level of miR‐17‐5p and reduced TXNIP expression after HI. Furthermore, miR‐17‐5p inhibition increased TXNIP expression and reversed the protective, anti‐apoptotic effects of GW0742.^11^ In this study, we focused on whether miR‐17‐5p can be regulated by PPAR‐β/δ. It has been previously shown that TXNIP expression can be regulated by PPAR‐α^59^ and PPAR‐γ.^9^ Here we hypothesized that PPAR‐β/δ can also regulate TXNIP expression—in a non‐direct manner, by activating miR‐17‐5p transcription. PPAR's can activate miRNA's by binding to PPAR response elements (PPRE) in promoter regions of miRNA's. While there is a plethora of examples of miRNAs‐dependent regulation of PPAR's in different cells and tissues,^19^ the information about PPAR's affecting miRNA levels is scarce. Most reports mention PPAR‐γ as a regulator of several miRNAs in distinct pathophysiological processes, including endothelial function, adipocytes differentiation, fibrosis, carcinogenesis and inflammation. When adult rats were treated with PPAR‐γ agonist rosiglitazone, expression of 28 miRNAs was changed significantly, with 12 of the miRNA's being up‐regulated in the cerebral cortex compared to controls.^21^ Some of those miRNA were shown to regulate PPAR‐γ, demonstrating a complicated feedback loop mechanism—miR‐145 and miR‐329 activated PPAR‐γ, which in turn modulated their expression. Moreover, it has been identified that up‐regulation of PPAR‐β/δ alleviated OGD‐induced miR‐15a expression in cerebral vascular endothelial cells^20^ and treatment of HUVEC endothelial cells with a PPAR‐β/δ agonist (GW501516) increased miR‐100 level and was vasculo‐protective.^60^ Here, we show that miR‐17‐5p expression can be regulated by PPAR‐β/δ. Cells transfected with the Luciferase vector and treated with GW0742 were showing higher miR‐17‐5p expression compared to vehicle‐treated control group transfected with the vector. Additionally, GW0742 treatment after OGD in our study resulted in inhibition of miR‐17‐5p target TXNIP in PC12 cells. Together with our previous findings showing that GW0742 treatment increased miR‐17‐5p levels in ipsilateral hemisphere after HI, our results are adding new insights to a complex regulatory network of neuroinflammation and pointing to another layer of regulation of NLRP3 by PPAR‐β/δ in HI brain injury. Our results indicate that miR‐17‐5p is an upstream therapeutic target for blocking TXNIP and NLRP3, and consequently inhibiting the neuroinflammation after HI. Direct binding of PPAR‐β/δ to the miR‐17 promoter was not investigated, and additional experiments are needed to confirm this regulation and exclude off‐target effects of pharmacological activation of PPAR‐β/δ by GW0742.

In conclusion, our findings demonstrated that PPAR‐β/δ activation by GW0742 reduces NLRP3‐related neuroinflammation after HI and in PC12 cells, and these effects are mediated by increased expression of miR‐17‐5p and inhibition of its target TXNIP. Therefore, GW0742 may provide a promising option for the management of HI patients.

## CONFLICT OF INTEREST

The authors confirm that there are no conflicts of interest.

## AUTHOR CONTRIBUTION

**Marcin Gamdzyk:** Conceptualization (equal); Data curation (equal); Methodology (equal); Writing‐original draft (equal); Writing‐review & editing (equal). **Desislava Met Doycheva:** Data curation (equal); Formal analysis (equal). **Ruiqing Kang:** Formal analysis (equal); Investigation (equal). **Hong Tang:** Data curation (equal). **Zackary D. Travis:** Methodology (equal). **Jiping Tang:** Project administration (equal); Supervision (equal). **John H. Zhang:** Conceptualization (equal); Project administration (equal); Supervision (equal).

## Supporting information

Fig S1‐S4

## Data Availability

The data that support the findings of this study are available from the corresponding author upon reasonable request.
