# Supplementary material for: GW0742 activates miR‐17‐5p and inhibits TXNIP/NLRP3‐mediated inflammation after hypoxic‐ischaemic injury in rats and in PC12 cells
Source: J Cell Mol Med. 2020 Oct 9;24(21):12318–30. doi: 10.1111/jcmm.15698 (PMC7686982; doi:10.1111/jcmm.15698)
Supplement: Supplementary file 1 — Fig S1‐S4 [file JCMM-24-12318-s001.pdf]

Supplementary information

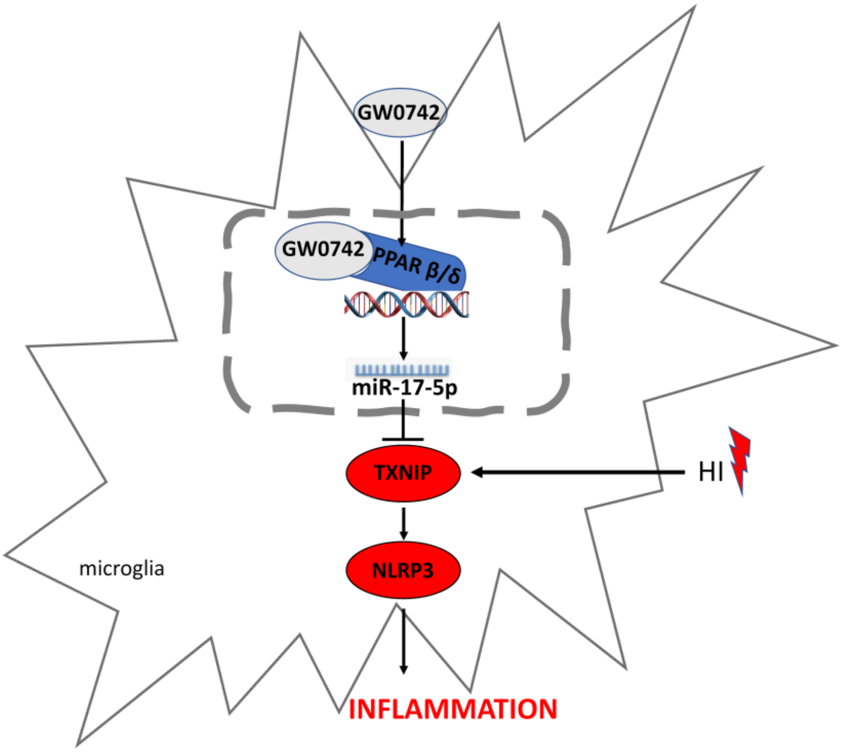

SUPPLEMENTARY FIGURE 1. Illustration of proposed mechanism.

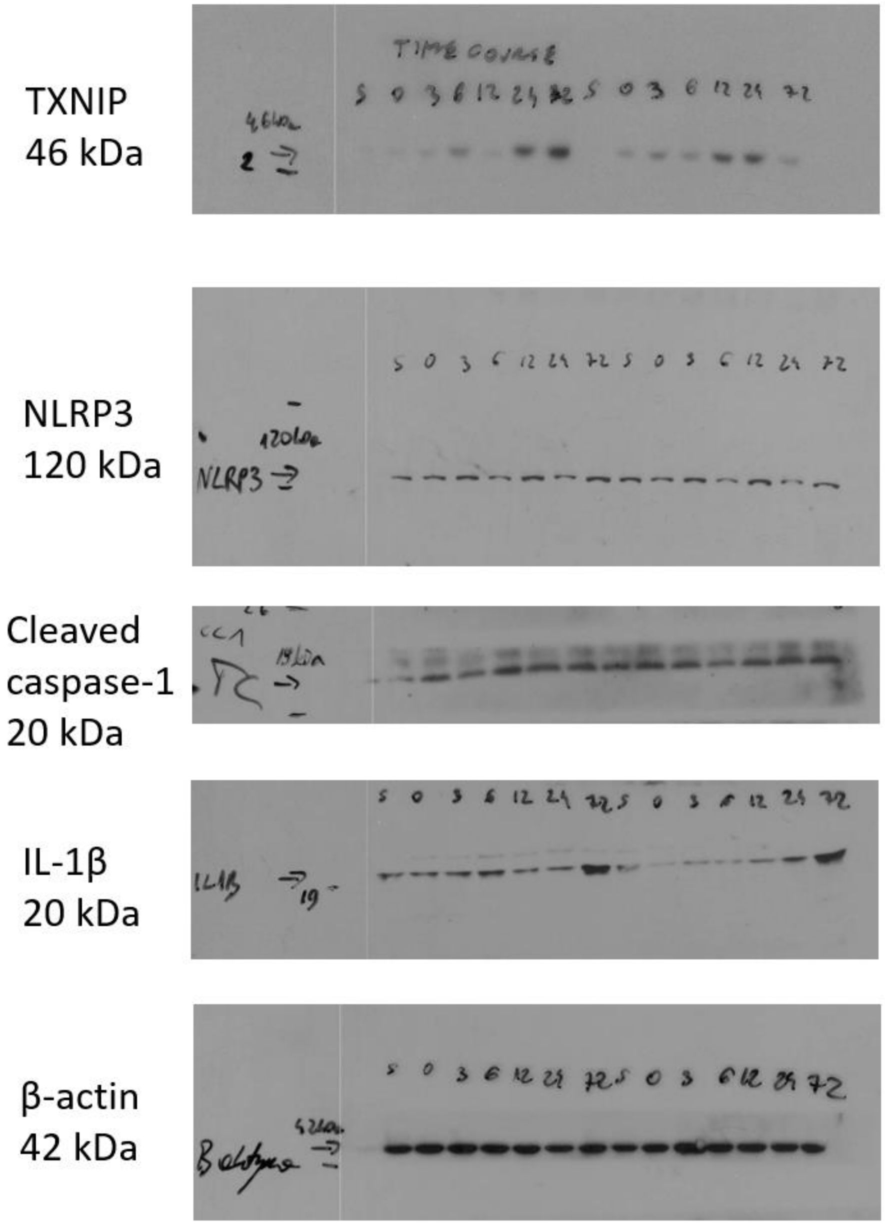

SUPPLEMENTARY FIGURE 2. Western blot membranes for time-course experiment shown in Fig.1.

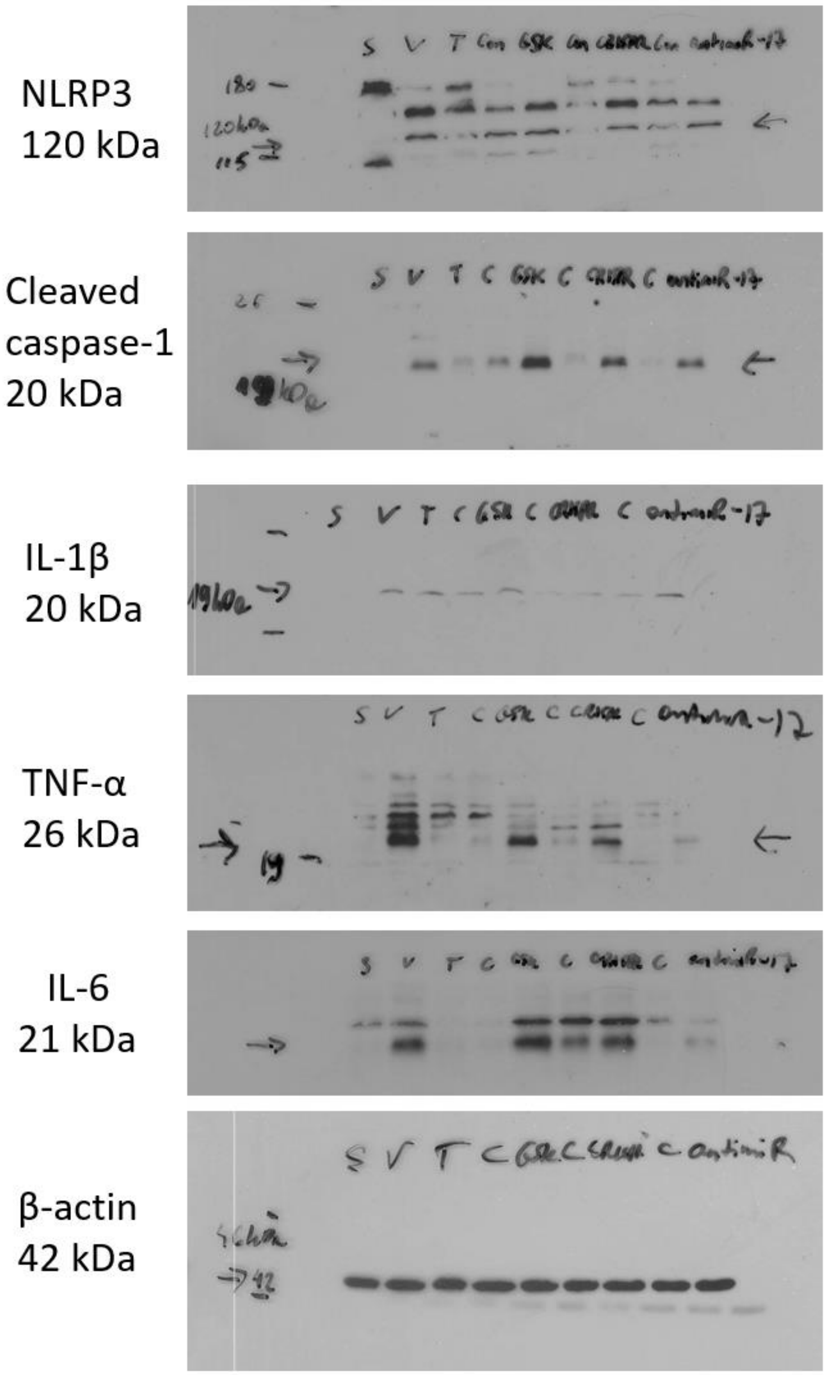

SUPPLEMENTARY FIGURE 3. Western blot membranes for mechanism study experiment shown in Fig.5.

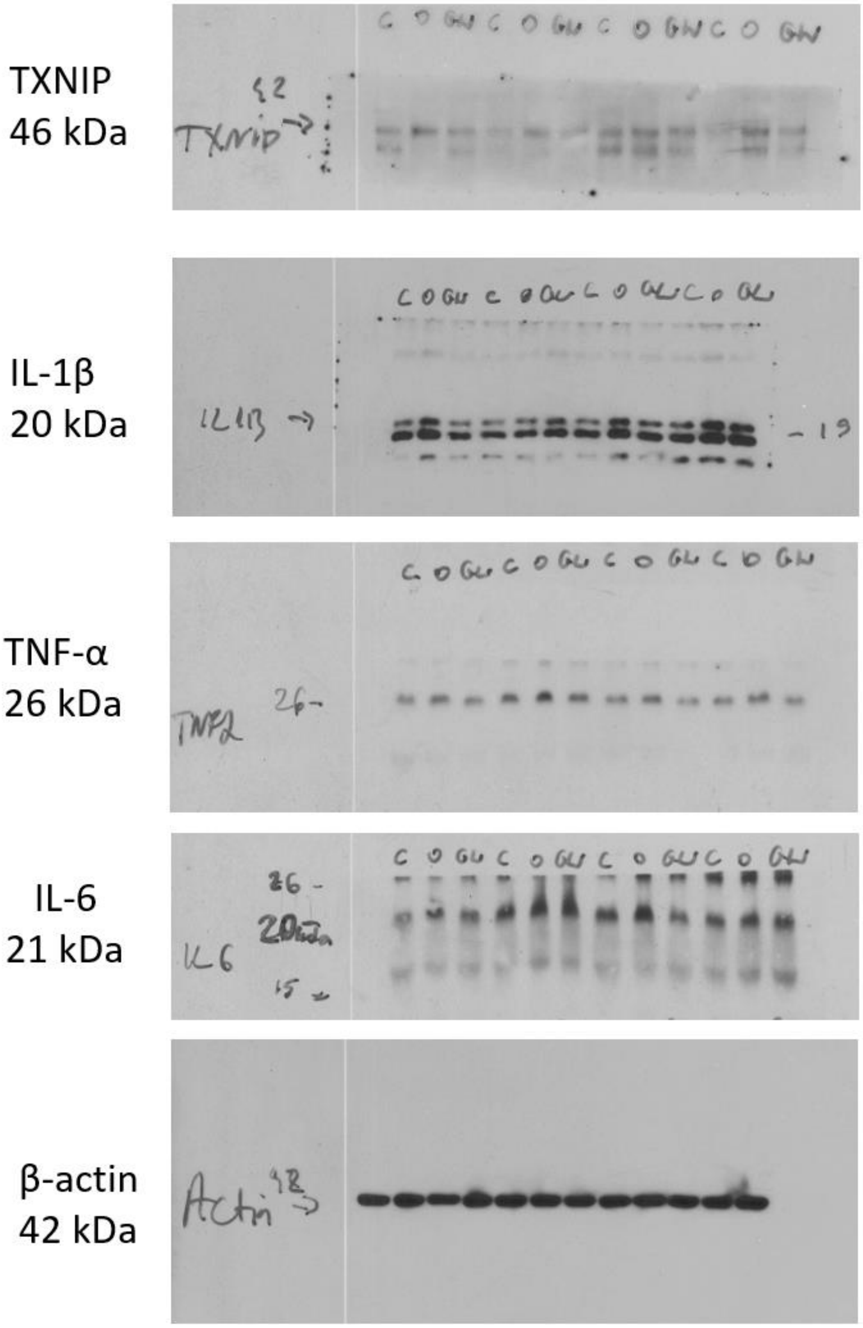

SUPPLEMENTARY FIGURE 4. Western blot membranes for PC12 cell culture experiment shown in figure 6.
